# A Novel Pathway for Metabolism of the Cardiovascular Risk Factor Homoarginine by alanine:glyoxylate aminotransferase 2

**DOI:** 10.1038/srep35277

**Published:** 2016-10-18

**Authors:** Roman N. Rodionov, Elisa Oppici, Jens Martens-Lobenhoffer, Natalia Jarzebska, Silke Brilloff, Dmitrii Burdin, Anton Demyanov, Anne Kolouschek, James Leiper, Renke Maas, Barbara Cellini, Norbert Weiss, Stefanie M. Bode-Böger

**Affiliations:** 1 University Center for Vascular Medicine, Technische Universität Dresden, Fetscherstraße 74, 01307 Dresden, Germany; 2 Department of Neuroscience, Biomedicine and Movement Sciences, Section of Biological Chemistry, University of Verona, Strada Le Grazie 8, 37134 Verona, Italy; 3 Institute of Clinical Pharmacology, Otto-von-Guericke University, Leipziger Str.44, 39120 Magdeburg, Germany; 4 Department of General Physiology, Saint-Petersburg State University, University Embankment, 7-9, 199034 Saint-Petersbug, Russia; 5 Institute of Highly Pure Biopreparations, 7 Pudozhskaya str., 197110 Saint-Petersburg, Russia; 6 Nitric Oxide Signalling Group, MRC Clinical Sciences Centre (CSC), Du Cane Road, W12 0NN, London; 7 Institute of Clinical Sciences (ICS), Faculty of Medicine, Imperial College London, Du Cane Road, W12 0NN, London; 8 Institute of Experimental and Clinical Pharmacology and Toxicology, Friedrich-Alexander-University Erlangen-Nürnberg (FAU), Fahrstr. 17, 91054 Erlangen, Germany

## Abstract

Low plasma concentrations of L-homoarginine are associated with an increased risk of cardiovascular events, while homoarginine supplementation is protective in animal models of metabolic syndrome and stroke. Catabolism of homoarginine is still poorly understood. Based on the recent findings from a Genome Wide Association Study we hypothesized that homoarginine can be metabolized by alanine:glyoxylate aminotransferase 2 (AGXT2). We purified human AGXT2 from tissues of AGXT2 transgenic mice and demonstrated its ability to metabolize homoarginine to 6-guanidino-2-oxocaproic acid (GOCA). After incubation of HepG2 cells overexpressing AGXT2 with isotope-labeled homoarginine-d4 we were able to detect labeled GOCA in the medium. We injected wild type mice with labeled homoarginine and detected labeled GOCA in the plasma. We found that AGXT2 knockout (KO) mice have higher homoarginine and lower GOCA plasma levels as compared to wild type mice, while the reverse was true for AGXT2 transgenic (Tg) mice. In summary, we experimentally proved the presence of a new pathway of homoarginine catabolism – its transamination by AGXT2 with formation of GOCA and demonstrated that endogenous AGXT2 is required for maintenance of homoarginine levels in mice. Our findings may lead to development of novel therapeutic approaches for cardiovascular pathologies associated with homoarginine deficiency.

Cardiovascular disease (CVD) remains the leading cause of mortality and morbidity in the world, and therefore warrants further research to identify novel markers and mediators of cardiovascular damage to facilitate early and precise identification of individuals at risk and allow the development of new therapeutic interventions. Recent epidemiological studies have demonstrated an association between low circulating levels of the endogenous nonproteinogenic amino acid L-homoarginine and an increased risk of cardiovascular and cerebrovascular events^1^,^2^,^3^. Furthermore, low levels of homoarginine have been shown to correlate with all-cause mortality in patients referred for coronary angiography^1^, diabetic patients on hemodialysis^1^, patients with peripheral arterial disease^4^, heart failure^5^ or stroke^6^, and in renal transplant recepients^7^, as well as in the general population^8^. Dietary supplementation of homoarginine in rodents demonstrated that homoarginine may play a protective role in experimental models of cardiovascular and cerebrovascular diseases^6^,^9^,^10^. The mechanisms of the potential protective effects of homoarginine are not entirely understood, but may include direct or indirect influence on nitric oxide production^11^,^12^ and blood clotting^13^.

L-homoarginine is an endogenous structural homologue of L-arginine, which is present in human plasma at a concentration around 2 μM^14^,^15^,^16^,^17^. Homoarginine is synthesized from lysine by L-arginine:glycine amidinotransferase (AGAT)^6^,^18^,^19^,^20^. An alternative activity of AGAT is production of guanidinoacetate, which is subsequently converted to creatine by guanidinoacetate N-methyltransferase (GAMT)^21^. In addition to direct conversion of lysine to homoarginine by AGAT, it has also been speculated that lysine might be converted to homoarginine in a three-step process, which is catalyzed by the enzymes of urea cycle^20^,^22^,^23^. Two pathways of homoarginine catabolism have been described. It has been shown that homoarginine can serve as a substrate of nitric oxide synthases (NOS), which converts it into homocitrulline^12^,^24^ and it has also been demonstrated that homoarginine can be catabolized to urea by argininase^22^,^25^.

In 2013 two independent Genome Wide Association Studies (GWAS) aimed at identifying the genetic determinants of plasma homoarginine concentrations were published^6^,^26^. The first study identified single-nucleotide polymorphisms in the gene coding for the AGAT enzyme as the only locus associated with plasma homoarginine concentration in humans. The results of the second study pointed at two other genes as associated with circulating homoarginine levels. One of them was carbamoyl phosphate synthetase I (CPS1), the rate limiting enzyme for the hepatic urea cycle and the second one was alanine:glyoxylate aminotransferase 2 (AGXT2)^27^. AGXT2 is a mitochondrial pyridoxal 5′-phosphate (PLP) dependent aminotransferase, which is predominantly expressed in the liver and kidneys^27^,^28^,^29^. AGXT2 has previously been shown to catalyse a number of reactions, the most intensely studied being regulation of systemic levels of beta-aminoisobutyric acid (BAIB, also abbreviated in the literature as BAIBA) and of the recently recognized cardiovascular risk factors asymmetric and symmetric dimethylarginines (ADMA and SDMA)^27^,^28^,^29^,^30^,^31^,^32^,^33^. No experimental evidence demonstrating a role for AGXT2 in homoarginine metabolism has been provided until now. Intrigued by the discrepancy between the GWAS studies described above and the possibility that homoarginine is a novel substrate for AGXT2 we designed the current study to test the hypothesis that AGXT2 metabolizes homoarginine *in vitro* and *in vivo*.

## Results

### Purification of human AGXT2

**Figure 1:**
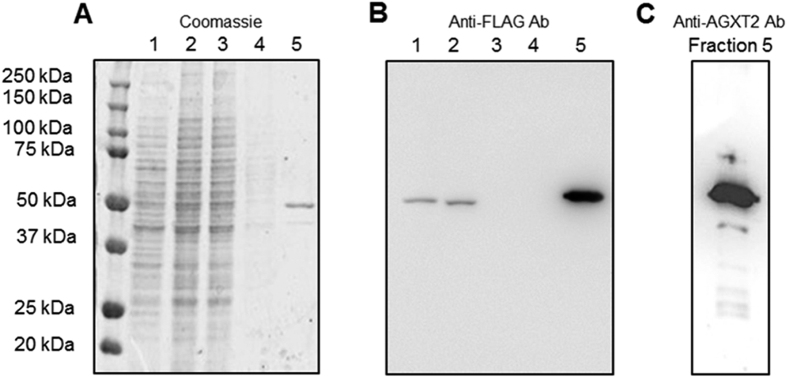
Purification of human AGXT2. Recombinant human AGXT2 was purified from tissues of transgenic mice with ubiquitous overexpression of human AGXT2 fused with a FLAG epitope at the C-terminus, by using an anti-FLAG affinity column. The identity of the protein was confirmed with both anti-FLAG and anti-AGXT2 antibodies. (**A**) Analysis of the purification steps by SDS-PAGE followed by staining with Coomassie blue. 1 – crude kidney homogenate, 2 – sample load, 3 – flow through, 4 – wash, 5 – elution. (**B**) Western Blot of the samples in (**A**) stained with the anti-FLAG antibody: 1 – crude kidney homogenate, 2 – sample load, 3 – flow through, 4 – wash, 5 – elution. (**C**) Western Blot of the elution fraction (corresponds to the lane 5 from the panels A and B) stained with the anti-AGXT2 antibody.

We purified recombinant human alanine:glyoxylate aminotransferase 2 (AGXT2) from tissue lysates of AGXT2 transgenic (AGXT2 Tg) mice. AGXT2 Tg mice that have been generated in our lab^28^ ubiquitously express human AGXT2 fused with a C-terminal FLAG-epitope under control of the CMV early enhancer/chicken β-actin (CAG) promoter and will be described in greater detail in a separate manuscript. The protein was purified by FLAG-affinity chromatography in a single-step procedure giving a yield of 0.15 mg/g of tissue. Coomassie blue-staining of SDS-PAGE gels containing protein aliquots from various steps of the purification process indicates that the final purified protein fraction is approximately 95% pure and displays an apparent molecular weight of 50–52 kDa similar to our previous observations^27^ (Fig. 1A). The finding that the purified protein is recognized by both anti-FLAG antibody and anti-human-AGXT2 antibody confirmed the identity of the purified enzyme (Fig. 1B,C respectively).

### Activity of recombinant AGXT2 towards alanine and homoarginine

**Figure 2:**
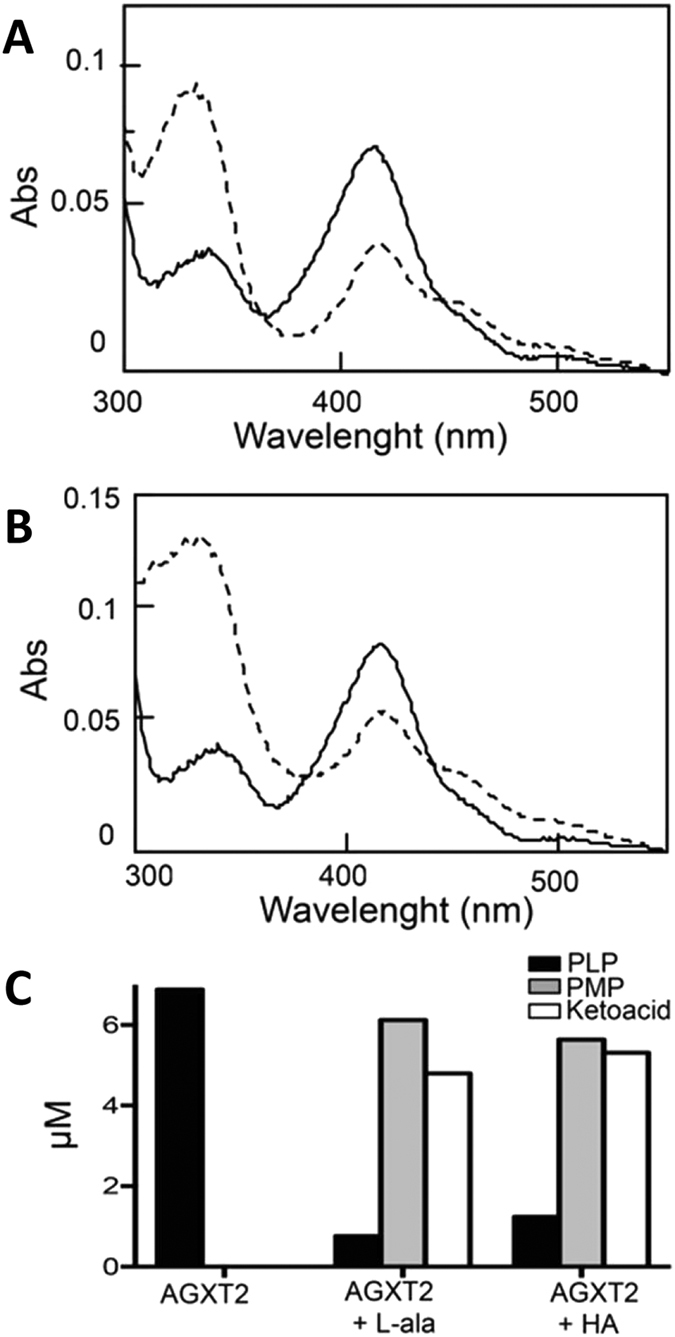
Tracing half-transamination of L-alanine and L-homoarginine by recombinant AGXT2 using spectrophotometric and HPLC analyses. (**A**) Absorbance spectrum of recombinant purified AGXT2 (0.4 mg/ml) in the absence (–) or in the presence (− −) of 30 mM L-alanine in 5 mM potassium phosphate buffer pH 8 + 0.15 M NaCl at 25 °C. (**B**) Absorbance spectrum of recombinant purified AGXT2 (0.4 mg/ml) in the absence (–) or in the presence (− −) of 30 mM L-homoarginine in 5 mM potassium phosphate buffer pH 8 + 0.15 M NaCl at 25 °C. (**C**) Coenzyme content of AGXT2 and ketoacid produced before and after incubation with L-alanine or L-homoarginine at 30 mM concentration. AGXT2 at 0.4 mg/ml concentration was incubated with either L-alanine or L-HA in 5 mM potassium phosphate buffer pH 8 + 0.15 M NaCl at 25 °C. After 2 min, the reaction was stopped by adding 10% (v/v) TCA and the amount of PLP, PMP, pyruvate and GOCA present in the mixture was determined by HPLC analysis.

**Figure 3:**
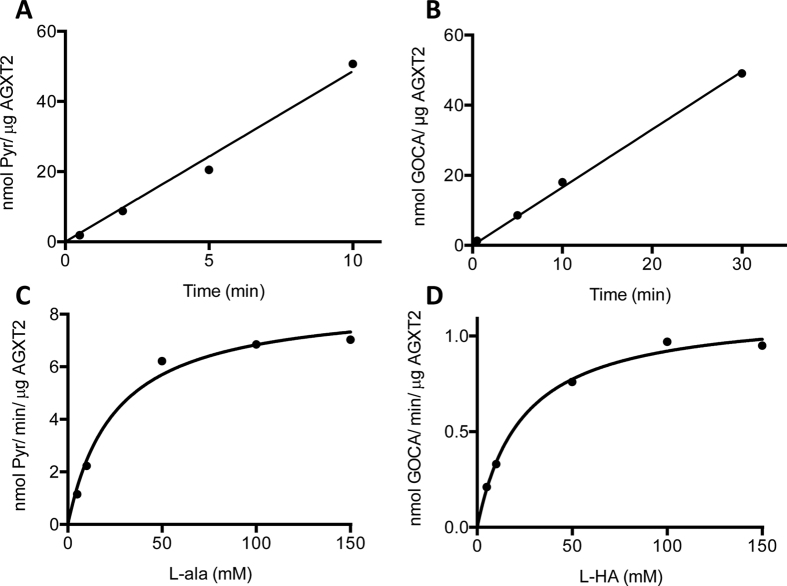
Kinetic analysis of the overall transaminase activity of recombinant purified AGXT2 for the L-alanine/glyoxylate and the L-homoarginine/pyruvate pairs. Recombinant purified AGXT2 (0.01 mg/ml) was incubated with either 30 mM L-alanine and 1 mM glyoxylate (**A**) or 30 mM L-homoarginine and 1 mM pyruvate (**B**) in the presence of 100 μM PLP in 5 mM potassium phosphate buffer pH 8 + 0.15 M NaCl at 25 °C. At various times, aliquots were withdrawn and the reaction was stopped by adding 10% (v/v) TCA. The amount of ketoacid produced was determined by HPLC analysis. The rate of the overall transamination was determined from the linear fit of the data. Panels (**C**,**D**) show the rate of the overall transamination as a function of L-alanine or L-homoarginine concentration, respectively. The line is derived from the theoretical fit to the Michaelis-Menten equation.

The absorbance spectrum of purified human AGXT2 displayed features typical of pyridoxal phosphate (PLP) dependent enzymes. Indeed, besides the band at 280 nm, the enzyme showed two bands in the visible region (at 337 and 414 nm) and a shoulder at 456 nm, indicative of the bound coenzyme. In order to understand, whether AGXT2 is able to perform the half-transamination of homoarginine we incubated the enzyme with either L-homoarginine or L-alanine (as a positive control) and followed the reaction using spectrophotometric and HPLC analyses. Upon incubation with 30 mM L-homoarginine or 30 mM L-alanine (Fig. 2A,B) the intensity of the 414 nm absorbance band decreased and a 332 nm band concomitantly appeared. These changes are indicative of a transamination reaction^34^. Accordingly, HPLC analyses revealed that in both cases 90% of PLP was converted to pyridoxyamine 5′-phosphate (PMP) (Fig. 2C) and that an equivalent amount of pyruvate and GOCA (the ketoacid produced by homoarginine half-transamination) is formed in the presence of L-alanine or L-homoarginine, respectively. Taken together, these data confirm that both L-alanine and L-homoarginine can be used by AGXT2 as amino donors. We therefore determined the kinetic parameters for the overall transamination of the L-alanine/glyoxylate and L-homoarginine/pyruvate pairs by measuring the amount of ketoacid produced over time (Fig. 3A,B) at various concentrations of amino donors (Fig. 3C,D). The results of these experiments indicate that AGXT2 displays a low *k*_cat_ for both substrate pairs and a K_m_ in the millimolar range (Table 1).

### Metabolism of homoarginine by AGXT2 in cultured hepatocytes

**Figure 4:**
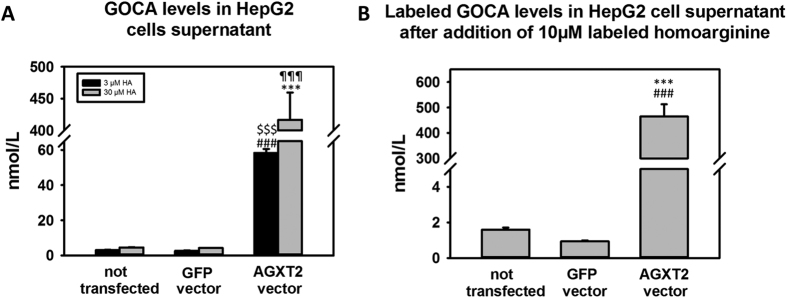
Detection of the product of homoarginine transamination by AGXT2 in cell culture system. (**A**) Levels of the homoarginine transamination product 6-guanidino-2-oxocaproic acid (GOCA) in Hep G2 cells supernatant after 24 h incubation with 3 μmol and 30 μmol of homoarginine. (**B**) Levels of the homoarginine transamination product 6-guanidino-2-oxocaproic acid (GOCA) in Hep G2 cells supernatant after 24 h incubation with 10 μmol of isotope-labeled homoarginine-d4. HA – homoarginine; Not transfected – cells only; GFP vector – cells transfected with GFP-encoding vector; AGXT2 vector – cells transfected with AGXT2 vector. (**A**) ^$$$^p < 0.001 vs 3 μM HA not transfected; ^###^p < 0.001 vs 3 μM HA GFP vector; ^¶¶¶^p < 0.001 vs 30 μM HA not transfected; ***P < 0.001 vs 30 μM HA GFP vector. (**B**) ***p < 0.001 vs GFP vector, ^###^p < 0.001 vs not transfected.

In order to determine the ability of AGXT2 to metabolize homoarginine in intact cells we overexpressed human AGXT2 in human hepatocytes (HepG2 cells) using an adenoviral vector (referred to as “AGXT2 vector”). An identical vector encoding GFP (referred to as “GFP vector”) served as a control. Upon addition of homoarginine to the cell culture medium we observed a significant, dose dependent increase in GOCA concentration in the medium (Fig. 4A, p < 0.001 AGXT2 vector vs GFP vector and p < 0.001 AGXT2 vector vs not transfected). We repeated this experiment using stable isotope-labeled L-homoarginine and observed small but detectable levels of labeled GOCA in the medium from control cells consistent with metabolism of labeled homoarginine by endogenous AGXT2. In AGXT2 transfected cells we observed a marked elevation of GOCA concentration in the medium in comparison to the cells transfected with the GFP vector (Fig. 4B, p < 0.001).

### Metabolism of homoarginine in wild type mice

**Figure 5:**
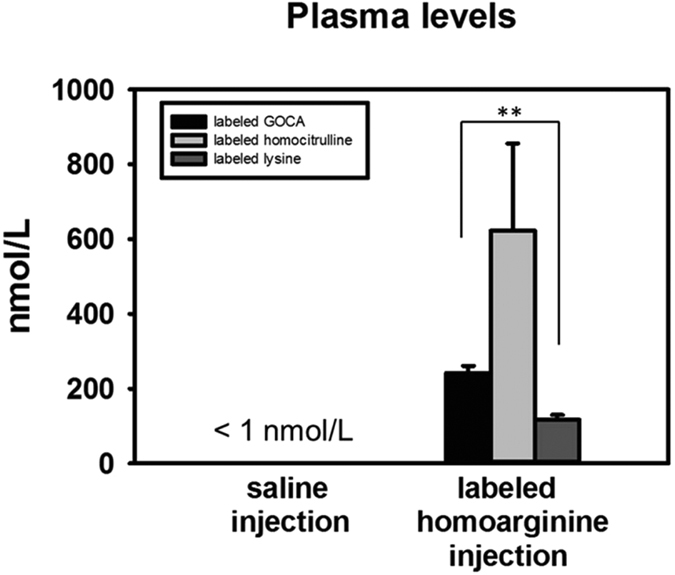
Labeled GOCA, homocitrulline and lysine levels in mice after injection of labeled homoarginine. Levels of isotope-labeled homoarginine transamination product 6-guanidino-2-oxocaproic acid (GOCA), labeled homocitrulline and labeled lysine in plasma of wild type mice 4 hours after i.p injection of 25 μmol/kg isotope-labeled homoarginine-d4. **P < 0.01

In order to determine the contribution of AGXT2 to homoarginine metabolism *in vivo*, we assessed plasma levels of stable isotope-labeled GOCA in C57/BL6 mice 4 hours after intraperitoneal injection of stable isotope-labeled L-homoarginine or saline. We were able to detect labeled GOCA in plasma of the mice injected with labeled L-homoarginine but not in plasma of saline injected control animals. In addition to metabolism to GOCA catalyzed by AGXT2 we were also able to detect the products of alternative pathways of homoarginine metabolism namely L-homocitrulline and L-lysine (Fig. 5).

### Homoarginine metabolism in AGXT2 knock-out and transgenic mice

**Figure 6:**
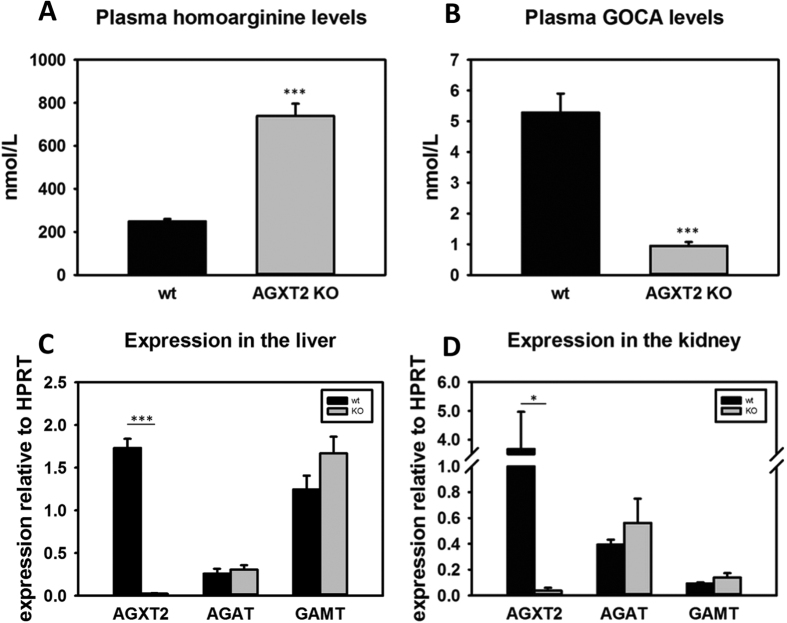
Homoarginine metabolism in AGXT2 knock-out mice. Plasma homoarginine (**A**) and 6-guanidino-2-oxocaproic acid (GOCA) (**B**) levels in wild type (wt) and alanine-glyoxylate aminotransferase 2 knock-out (AGXT2 KO) mice. ***P < 0.001 vs wt. mRNA levels of alanine-glyoxylate aminotransferase 2 (*Agxt2*), L-arginine: glycine amidinotransferase (*Agat*) and guanidinoacetate N-methyltransferase (*Gamt*) in the liver (**C**) and kidney (**D**) of AGXT2 KO and wt mice. ***P < 0.001; *P < 0.05.

**Figure 7:**
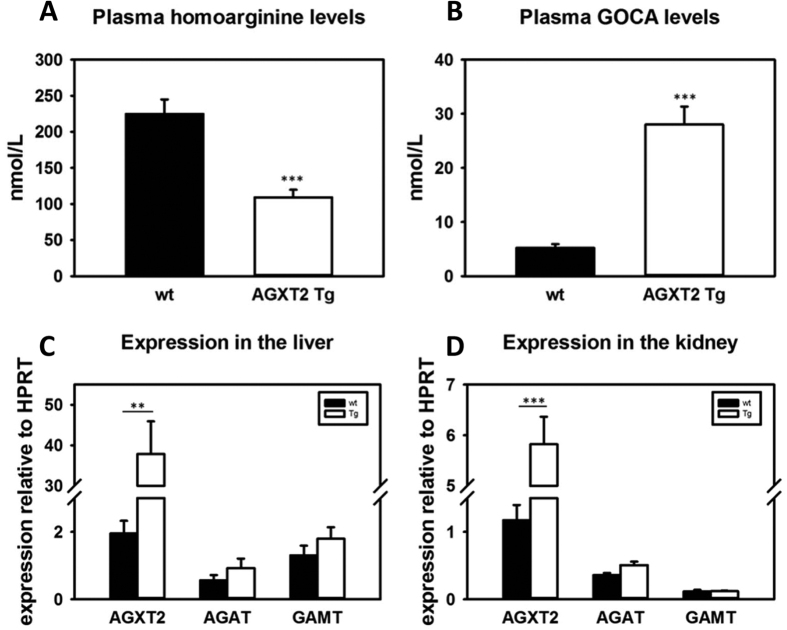
Homoarginine metabolism in AGXT2 transgenic mice. Plasma homoarginine (**A**) and 6-guanidino-2-oxocaproic acid (GOCA) (**B**) levels in wild type (wt) and alanine-glyoxylate aminotransferase 2 transgenic (AGXT2 Tg) mice; ***P < 0.001 vs wt. mRNA levels of alanine-glyoxylate aminotransferase 2 (*Agxt2*), L-arginine: glycine amidinotransferase (*Agat*) and guanidinoacetate N-methyltransferase (*Gamt*) in the liver (**C**) and kidney (**D**) of AGXT2 Tg and wt mice. ***P < 0.001; **P < 0.01.

Next we characterized homoarginine metabolism in genetically modified mouse models of AGXT2 deficiency (AGXT2 KO) and overexpression (AGXT2 Tg). We measured plasma homoarginine levels in AGXT2 KO mice and found that they were increased 3-fold in comparison to their wild-type littermates (Fig. 6A, p < 0.001). Correspondingly, GOCA levels were 6-fold higher in wild type mice comparing to AGXT2 KO mice (Fig. 6B, p < 0.001). In agreement with these data, plasma homoarginine levels in AGXT2 Tg mice were two fold lower (Fig. 7A, p < 0.001) and GOCA levels five fold higher (Fig. 7B, p < 0.001) than that of wild type animals. To test whether the observed changes in plasma homoarginine and GOCA levels in the AGXT2 deficient and overexpressing mice were attributable solely to the reduction or elevation of AGXT2, we performed RT-PCR with primers specific for other enzymes involved in the metabolism of homoarginine. We did not detect any changes in the mRNA levels of arginine:glycine amidinotransferase (AGAT) and guanidinoacetate N-methyltransferase (GAMT) in the livers and kidneys of AGXT2 KO or Tg mice as compared to the wild type littermates (Figs 6C,D and 7C,D, p > 0.05).

## Discussion

**Figure 8:**
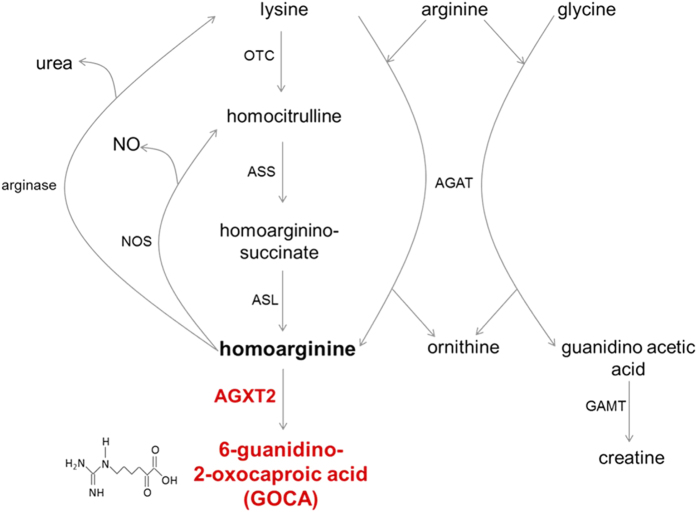
Updated homoarginine metabolism pathway. AGXT2 – alanine:glyoxylate aminotransferase 2; AGAT – arginine:glycine amidinotransferase; GAMT – guanidinoacetate N-methyltransferase; OTC – ornithine transcarbamyolase; ASS – argininosuccinate synthase; ASL – argininosuccinate lyase; NOS – nitric oxide synthase.

Homoarginine is an endogenous nonproteinogenic amino acid, which has recently been proposed to act as a protective factor in cardiovascular and renal diseases^35^,^36^,^37^. Hence, there is considerable interest in the identification of potential targets for pharmacological augmentation of homoarginine levels. In the 1960s it was discovered that homoarginine could be produced from L-lysine^38^ and that this reaction is catalyzed by AGAT (Fig. 8). Davids and colleagues reported that cultured human lymphoblasts from a patient with *AGAT* deficiency could not convert lysine to homoarginine, thus demonstrating relevance of this pathway in humans^20^. Deficiency of *Agat* in mice leads to a dramatic decrease in systemic homoarginine levels, indicating a significant role for this enzyme in regulation of homoarginine homeostasis^6^. An additional and indirect pathway for conversion of lysine to homoarginine has also been suggested in the literature. This pathway includes conversion of lysine to homocitrulline by ornithine transcarbamylase (OTC), conversion of homocitrulline to homoargininosuccinate by argininosuccinate synthase (ASS) and finally conversion of homoargininosuccinate to homoarginine by argininosuccinate lyase (ASL)^23^,^39^. However, the significance of the OTC-ASS-ASL pathway in homoarginine production has been questioned by Davids and coauthors, who reported that homoarginine concentrations were increased rather than decreased in a child with ASS deficiency.

The further metabolic fate of homoarginine remains to a large extent unknown. It was reported that homoarginine could be utilized by all three purified NOS isoforms as a substrate^12^,^40^. However, the Km of NOS for homoarginine is significantly higher than that for arginine and this observation, together with the significantly lower concentrations (approximately 30 fold lower) of homoarginine compared to arginine in biological fluids and tissues, make it unclear to what extent NOS could be contributing to homoarginine clearance. Another proposed pathway of homoarginine catabolism is its conversion to urea and lysine by L-arginase. Interestingly, in our study we were able to detect labeled homocitrulline and lysine in mouse plasma after i.p. injection of labeled homoarginine (Fig. 5). Our experimental design, however, does not allow us to distinguish between a) conversion of homoarginine to lysine by arginase and subsequent conversion of lysine to homocitrulline by OTC and b) conversion of homoarginine to homocitrulline by NOS and subsequent conversion of homocitrulline to lysine by OTC.

Prompted by associations between polymorphisms in AGXT2 and homoarginine levels reported in GWAS studies^26^ we have performed experiments aimed at determining the role of AGXT2 in homoarginine metabolism *in vitro* and *in vivo*. Initially we purified recombinant human AGXT2 from tissues of AGXT2 Tg mice (Fig. 1) to determine if it could utilize L-homoarginine as a substrate. Absorbance spectroscopy and HPLC studies confirmed that the purified enzyme was active and was able to bind PLP and to catalyse the overall transamination of the L-alanine/glyoxylate pair, in agreement with previous results^41^. Furthermore, our data clearly demonstrated that the enzyme can utilize L-homoarginine as an amino donor (Figs 2 and 3). However, the catalytic efficiency measured *in vitro* is relatively low, mainly due to a high K_m_ value (Table 1). Previous enzymology studies of rat AGXT2 reported that the enzyme displays *in vitro* K_m_ values of about 0.13, 8, 10 and 13 mM for BAIB, L-alanine, ADMA and SDMA, respectively using pyruvate or glyoxylate as amino acceptors^32^. These data seem to indicate that the preferred substrate of AGXT2 is BAIB and this suggestion is also supported by the observation that in humans AGXT2 deficiency leads to hyper-beta-aminoisobutyric aciduria. However, a significant role for AGXT2 in ADMA and SDMA catabolism has also been repeatedly demonstrated *in vivo* despite the apparently low *in vitro* catalytic efficiency of AGXT2 towards these substrates. Thus, mice with targeted deletion of AGXT2 or adenovirus-mediated overexpression have significantly elevated or reduced plasma concentrations of both methylarginines respectively^27^,^33^,^42^. The finding that the K_m_ for homoarginine of human AGXT2 is of the same order of magnitude of those of ADMA and SDMA suggests that AGXT2 could also play a role in homoarginine metabolism *in vivo*.

In order to extend or biochemical observations to intact cells and whole animals we analysed the ability of AGXT2 to utilize homoarginine as a substrate in cultured cells and *in vivo* in animal models and found out that both human AGXT2 in HepG2 cells and mouse AGXT2 *in vivo* can indeed metabolize homoarginine with formation of the corresponding keto-acid, which we abbreviated as GOCA (Fig. 4A,B). Interestingly, injection of labelled L-homoarginine in mice resulted in plasma levels of labelled GOCA that were of the same order of magnitude as the levels of the other labelled homoarginine metabolites L-homocitrulline and L-lysine (Fig. 5) suggesting that that, at least under these experimental conditions, the contribution of AGXT2 to the enzymatic clearance of L-homoarginine is comparable to previously characterised homoarginine metabolic pathways that comprise the arginase/NOS enzymes (Fig. 8).

Finally, we observed that plasma homoarginine concentrations were elevated in *Agxt2* KO mice (Fig. 6A) and decreased in AGXT2 Tg mice (Fig. 7A), while the plasma concentration of GOCA showed reciprocal changes. (Figs 6B and 7B). Interestingly, expression of the other major enzymes involved in homoarginine metabolism (AGAT & GAMT) were unchanged both in *Agxt2* KO and AGXT2 Tg mice (Figs 6C,D and 7C,D), suggesting that the observed changes in circulating levels of homoarginine and GOCA are the result of changes in AGXT2 expression. These data provide the first evidence that at least in mice endogenous AGXT2 is required for maintenance of systemic homoarginine levels. In the LURIC cohort the homoarginine plasma concentration of the individuals, who were heterozygous or homozygous for the AGXT2 rs37369 T-allele, was elevated by 8% and 12%, respectively^26^. Together with our experimental data this suggests that AGXT2 might also be relevant for the homoarginine homeostasis in humans.

Our current findings increase the number of the known biologically active substrates of AGXT2, and suggest that the enzyme might play a role in the metabolism of several distinct substrates that each have their own physiological and pathophysiological roles. It has been reported that about 1–3% of the European and up to 50% of the Asian populations are homozygous for AGXT2 polymorphisms, which lead to partial reduction of AGXT2 activity and result in the biochemical trait hyper-beta-aminoisobutyric aciduria^43^. The physiological consequences of AGXT2 deficiency in humans are still poorly understood. *Agxt2* deficient mice are viable and develop mild hypertension presumably due to elevation of systemic ADMA levels^33^. Our discovery of the role of AGXT2 in metabolism of homoarginine together with the suggested protective role of homoarginine in cardiovascular disease states may explain why in some areas, such as Japan, up to 50% of the population show a functional AGXT2 deficiency without evidence for excess cardiovascular mortality, as it might have been expected from the loss of AGXT2-mediated clearance of ADMA. Further studies will be required to understand the net effect AGXT2 deficiency in general population as well as in specific subgroups of patients with ADMA- or homoarginine-associated pathologies. Inhibition of AGXT2 might be an attractive therapeutic approach for those pathologies and for those populations, in which the beneficial role of homoarginine would overweigh the potential negative effects of elevation of the other potentially hazardous AGXT2 substrates, such as ADMA and SDMA.

## Summary and Future Perspectives

In the presented work we identified a previously unknown pathway of homoarginine catabolism. We successfully purified for the first time human AGXT2 from tissues of AGXT2 Tg mice and showed that the recombinant enzyme can metabolize L-homoarginine to GOCA. We were also able to demonstrate that this reaction can take place both in cultured human cells and in *in vivo* in mice. Finally, we showed that endogenous AGXT2 is involved in the maintenance of systemic homoarginine levels in mice.

Clinical studies have demonstrated that low plasma levels of homoarginine are a risk factor for renal and cardiovascular diseases, as well as an independent predictor for all-cause mortality. It is still unclear, to what extent homoarginine itself may directly serve a protective role in cardiovascular disease and to what extent low homoarginine levels may be simply a marker of the concurrent changes in related metabolic pathways. Our discovery of homoarginine degradation by AGXT2 opens a new direction in research into the role of homoarginine as a marker and potentially a direct protective factor in human diseases. Furthermore, our current study broadens the range of potential therapeutic approaches for diseases associated with low circulating homoarginine levels.

## Materials and Methods

### Purification of human recombinant AGXT2 enzyme

Recombinant human AGXT2 was purified from the tissues of transgenic mice with ubiquitous overexpression of human AGXT2 fused with a FLAG epitope on the C-terminus (manuscript in preparation). All the purification steps were carried out at 0–4 °C unless differently stated. Frozen kidney and liver tissues of mice (18 g total weight) were collected and washed two times with 10 ml of homogenization buffer (HB) (5 mM potassium phosphate, pH 7.4 + 0.15 M NaCl + 10% glycerol + 0.1 mM pyridoxal phosphate). Tissues were then placed in a 50 ml falcon tube, cut with sterile surgical scissors and washed two times with 15 ml of HB to remove blood. The obtained sample was then placed in a beaker containing HB + a protease inhibitor cocktail (CompleteMini, Roche) in a final volume of 30 ml, homogenized with a blender and centrifuged at 10000 *g* for 30 min. The resulting pellet was frozen at −80 °C, thawed, resuspended in 30 ml of HB + protease inhibitor cocktail and sonicated 30 seconds at 70% maximal power four times. The insoluble material was removed by centrifugation at 10000 *g* for 30 min and the supernatant was rapidly heated at 60 °C for 3 min. After centrifugation of the mixture at 13000 *g* for 30 min the supernatant was filtered through sterile dressing and loaded in a 1 ml hand-packed anti-FLAG affinity column (ANTI-FLAG M2 affinity gel, Sigma-Aldrich). The column was then washed with 20 ml of HB and the bound AGXT2-FLAG protein was eluted by the addition of 5 ml of HB containing 100 μg/ml FLAG peptide (Sigma-Aldrich). The eluted protein was concentrated by forced dialysis via an Amicon device (Millipore) and stored at −20 °C.

### Western Blot for recombinant AGXT2

Anti-human AGXT2 antibody was from Sigma-Aldrich, Munich, Germany (cat. No. HPA037382).

Twenty micrograms of samples deriving from each protein purification step or 0.5 μg of purified AGT2-FLAG were loaded on a Mini Protean TGX^TM^ pre-cast gel (Biorad) along with the Precision plus protein Kaleidoscope^TM^ (Bio-Rad) molecular mass markers. The gel was either stained with Coomassie brilliant blue or transferred to a nitrocellulose membrane by an iBlot device (Invitrogen). The membrane was then blocked in 5% milk for 1 h at 37 °C. For AGXT2-FLAG detection the membrane was incubated with 1:2000 monoclonal mouse anti-FLAG antibody (Sigma-Aldrich) or with 1:500 rabbit anti-human AGT2 antibody (Sigma-Aldrich), washed three times in TBST (50 mM Tris–HCl pH 7.5, 150 mM NaCl, 0.1% Tween 20) and then incubated with 1:2000 peroxidase-conjugated anti mouse IgG or 1:6000 peroxidase-conjugated anti rabbit IgG. Blotted proteins were detected with the ECL^®^ reagent (Millipore), using a ChemiDoc XRS Imaging System (Bio-Rad, Hercules, CA).

### Kinetic studies

To monitor the half-transaminase activity 250 μl of purified AGXT2 (0.4 mg/ml) were incubated either in the presence or in the absence of L-alanine or L-homoarginine (30 mM) in 5 mM potassium phosphate (KP) buffer pH 8 + 0.15 M NaCl at 25 °C. After 30 min the reaction was stopped by adding 10% (v/v) trichloroacetic acid (TCA). The production of pyridoxal 5′-phosphate (PLP) and pyridoxamine 5′-phosphate (PMP) was determined by a previously described HPLC method The produced ketoacid was quantified by high-performance liquid chromatography (HPLC) upon derivatization with 2,4-dinitrophenylhydrazine as previously described^44^. Standard curves with known amounts of PLP, PMP, and pyruvate were prepared prior to each analysis.

The rate of the overall transamination reaction of purified AGXT2 (0.01 mg/ml) for the pair L-alanine/glyoxylate and L-homoarginine/pyruvate was determined in potassium phosphate (KP) buffer pH 8 + 0.15 M NaCl + 200 μM pyridoxal 5′-phosphate (PLP) in the presence of 33 mM amino-donor and 1 mM amino-acceptor at 25 °C. At different times (2, 5, 10 and 30 min), 30 μl of each mixture were withdraw and the reaction was stopped by adding 10% (v/v) TCA. The amount of the ketoacid produced was determined by HPLC as previously described^44^. By varying the amino donor concentration (5, 10, 50, 100 and 150 mM) at a fixed amino acceptor concentration (1 mM), the kinetic parameters of purified AGXT2 (0.01 mg/ml) for the pair L-alanine/glyoxylate and L-homoarginine/pyruvate were determined. Data of initial velocity (v) as a function of substrate concentration were fitted to the Michaelis-Menten equation:


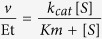


where Et is the total enzyme concentration, *S* is the substrate concentration, *k*_*cat*_ is the turnover number, and *Km* is the Michaelis-Menten constant.

### Animals

All animal protocols were approved by the animal protection authority of Saxony, Germany and all the experiments were carried out in accordance with the approved guidelines. Mice had unlimited access to food and water and were kept in 12 h light/darkness cycle. The alanine glyoxylate aminotransferase 2 transgenic (AGXT2 Tg) mice with ubiquitous overexpression of human AGXT2 fused with FLAG epitope on the C-terminus were generated in our laboratory (manuscript in preparation). The mice were genotyped with the following primer pair, which is specific for the human transgene: forward 5′ GTTGGCAGAGGCAGCATT 3′ and reverse 5′ GTCGTCATCCTTGTAATCCTTAGC 3′. The alanine glyoxylate aminotransferase 2 transgenic deficient (AGXT2 KO) mice have been described previously and were genotyped with the following primer pairs: forward 5′ GCATAATGTCCTGCCTTTCGGAG 3′, reverse 5′ CCCTAGGAATGCTCGTCAAGA (knock-out littermates) and 5′ GCATAATGTCCTGCCTTTCGGAG 3′, reverse 5′ CAGCAATTGATGTGGCCACAGAAG 3′ (wild-type littermates).

### Collection of tissue samples

Animals were sacrificed and blood was collected by cardiac puncture into EDTA containing tubes (final concentration 5 mmol/L). Plasma was separated by centrifugation and stored at −80 °C. Kidney and liver samples were collected and flash-frozen immediately after the sacrifice of the animals and stored at −80 °C until further analysis.

### Injection of labeled homoarginine in mice

Isotope-labeled homoarginine-d4 (Toronto Chemicals Research) was injected i.p. in 4 wild-type C57BL/6 mice in a dose of 25 μmol/kg. Four control mice received injection of saline. 4 hours later the mice were sacrificed and plasma samples were collected.

### Measurement of homoarginine, 6-guanidino-2-oxocaproic acid (GOCA) and homocitrulline in cell supernatant and mice plasma

Measurements of L-homoarginine were conducted by LC-MS/MS according to a previously described procedure^45^. The determination of 6-guanidino-2-oxocaproic acid (GOCA) was performed by LC-MS/MS in a similar way as the structurally closely related ADGV (also abbreviated as DMGV)^46^. GOCA was unambiguously identified in the samples by comparison with an authentic standard (Synthon-Lab, Sankt Petersburg, Russia). The quantification of labeled D_4_-homocitrulline and D_4_-lysine in plasma was performed by LC-MS/MS. Sample preparation and chromatographic separation was carried out according to a previously described procedure Mass spectrometric detection was achieved by adaption of the settings described in ref. 47. Internal standard for quantification of D_4_-homocitrulline was unlabeled homocitrulline at a concentration of 10 μmol/L, corrected for the endogenous amount of this substance in the samples. The internal standard for D_4_-lysine was ^13^C_6_-^15^N_2_-lysine at a concentration of 10 μmol/L.

### Real-time polymerase chain reaction (RT-PCR)

Messenger RNA (mRNA) levels were determined by real-time reverse transcription polymerase chain reaction (RT-PCR). The RNA isolation from tissues was carried out using the NucleoSpi RNa kit (Macherey-Nagel, Düren, Germany). Reverse transcription of the isolated RNA to cDNA was achieved by applying the RevertAid First Strand cDNA Synthesis kit (Fermentas, Fisher Scientific, Schwerte, Germany). The amplification of the cDNA templates for quantification was carried out using the Maxima SYBR Green/Rox mix (Thermo Scientific, Germany). DNA was first denaturated for 10 min at 95 °C, followed by 40 cycles of denaturation for 15 s at 95 °C and annealing and extending for 60 s at 60 °C. Data were analyzed using 7500 software (version 2.0.5, Applied Biosystems) and expressed as a ratio to levels of hypoxanthine-guanine-phosphoribosyltransferase (HPRT) mRNA. The following primer pairs were used: (HPRT) forward 5′ CTTTGCTGACCTGCTGGATTAC 3″ and reverse 5′; ATCCAACACTTCGAGAGGTCC 3′; arginine:glycine amidinotransferase (AGAT) forward 5′ CAATGGCTGACGAACTGTAT 3′ and reverse 5′ TGTAACCTGGCTTCTCTGT 3′; guanidinoacetate N-methyltransferase (GAMT) forward 5′ TGGCACACTCACCAGTTCA 3′ and reverse 5′ AAGGCATAGTAGCGGCAGTC 3′; alanine glyoxylate amninotransferase 2 (AGXT2) forward 5′ TGGGCTCTCACTTCTGGG 3′ and reverse 5′′ CACCTCAAGCACAGCAGAT 3′ (for tissues from AGXT2 transgenic mice) and forward 5′ GTTGTGACCACTCCAGAA 3′ and reverse 5′ TATCTTTTGAACCATCTC 3′ (for tissues from AGXT2 knock-out mice). The primers oligomers were synthesized by biomers.net (Germany).

### Statistical analysis

Statistical analysis was performed using SigmaPlot 12.0 and GraphPad Prism 5. Comparisons between the groups were done using two-tailed unpaired student t-tests and between more than two groups with one-way ANOVA. Statistical significance was defined as a P value <0.05. Values are reported as mean ± standard error of the mean.

## Additional Information

**How to cite this article**: Rodionov, R. N. *et al.* A Novel Pathway for Metabolism of the Cardiovascular Risk Factor Homoarginine by alanine:glyoxylate aminotransferase 2. *Sci. Rep.* **6**, 35277; doi: 10.1038/srep35277 (2016).

## Figures and Tables

**Table 1:** Steady-state kinetic parameters of recombinant AGXT2 for the L-alanine/glyoxylate and L-homoarginine/pyruvate pairs.

Substrate	Cosubstrate	K_m_ L-ala (mM)	K_m_ L-HA (mM)	*k*_cat_ (min^−1^)	*k*_cat_/K_m_ (min^−1^/mM^−1^)
L-alanine	Glyoxylate	25 ± 3		8.5 ± 0.5	0.34 ± 0.04
L-homoarginine	Pyruvate		24 ± 3	1.13 ± 0.04	0.047 ± 0.006
